# Walnut Prevents Cognitive Impairment by Regulating the Synaptic and Mitochondrial Dysfunction via JNK Signaling and Apoptosis Pathway in High-Fat Diet-Induced C57BL/6 Mice

**DOI:** 10.3390/molecules27165316

**Published:** 2022-08-20

**Authors:** Jong Hyun Moon, Jong Min Kim, Uk Lee, Jin Yong Kang, Min Ji Kim, Hyo Lim Lee, Hye Rin Jeong, Min Ji Go, Hyun-Jin Kim, Hye Won Park, Chul-Woo Kim, Sung Jin Park, Ho Jin Heo

**Affiliations:** 1 Division of Applied Life Science (BK21), Institute of Agriculture and Life Science, Gyeongsang National University, Jinju 52828, Korea; 2 Division of Special Forest Resources, Department of Forest Bio-resources, National Institute of Forest Science (NIFoS), Suwon 16631, Korea; 3 World Institute of Kimchi an Annex of Korea Food Research Institute, Gwangju 61755, Korea; 4 Research Institute for Advanced Industrial Technology, Korea University, Sejong 30019, Korea

**Keywords:** walnut, *Juglans regia*, high-fat diet, insulin resistance, inflammation, cognitive function, JNK/NFκB pathway

## Abstract

This study was conducted to evaluate the protective effect of *Juglans regia* (walnut, Gimcheon 1ho cultivar, GC) on high-fat diet (HFD)-induced cognitive dysfunction in C57BL/6 mice. The main physiological compounds of GC were identified as pedunculagin/casuariin isomer, strictinin, tellimagrandin I, ellagic acid-O-pentoside, and ellagic acid were identified using UPLC Q-TOF/MS analysis. To evaluate the neuro-protective effect of GC, 3-(4,5-dimethylthiazol-2-yl)-2,5-diphenyltetrazolium bromide (MTT), 2′,7′-dichlorodihydrofluorecein diacetate (DCF-DA) analysis were conducted in H_2_O_2_ and high glucose-induced neuronal PC12 cells and hippocampal HT22 cells. GC presented significant cell viability and inhibition of reactive oxygen species (ROS) production. GC ameliorated behavioral and memory dysfunction through Y-maze, passive avoidance, and Morris water maze tests. In addition, GC reduced white adipose tissue (WAT), liver fat mass, and serum dyslipidemia. To assess the inhibitory effect of antioxidant system deficit, lipid peroxidation, ferric reducing antioxidant power (FRAP), and advanced glycation end products (AGEs) were conducted. Administration of GC protected the antioxidant damage against HFD-induced diabetic oxidative stress. To estimate the ameliorating effect of GC, acetylcholine (ACh) level, acetylcholinesterase (AChE) activity, and expression of AChE and choline acetyltransferase (ChAT) were conducted, and the supplements of GC suppressed the cholinergic system impairment. Furthermore, GC restored mitochondrial dysfunction by regulating the mitochondrial ROS production and mitochondrial membrane potential (MMP) levels in cerebral tissues. Finally, GC ameliorated cerebral damage by synergically regulating the protein expression of the JNK signaling and apoptosis pathway. These findings suggest that GC could provide a potential functional food source to improve diabetic cognitive deficits and neuronal impairments.

## 1. Introduction

Diabetes, one of the major metabolic diseases, has increased in prevalence over the past few decades, and more than 150 million people currently suffer from diabetes worldwide [1]. The causes of diabetes are reported to obesity, hyperglycemia, insulin resistance, and hormone changes derived from changes in diet such as high-fat and high-carbohydrate diets [2]. A high-fat diet (HFD) impairs energy homeostasis in the whole body due to comprehensive factors and lifestyle changes and contributes to obesity [3]. Obesity is related to type 2 diabetes by causing insulin resistance, increasing fasting plasma insulin levels, and impairing glucose tolerance [4]. Hyperglycemia by glucose intolerance leads to an increase in the formation of advanced glycation end products (AGEs) and activation of nuclear factor-kappa B (NFκB), leading to the activation of inflammation [5]. Furthermore, brain tissue is easily damaged by the overproduction of reactive oxygen species (ROS) due to the metabolic abnormalities accompanying insulin resistance. Oxidative stress caused by HFD leads to the activation of c-Jun N-terminal kinase (JNK), which precedes cell death by apoptosis and inflammation through the downregulation of protein kinase B (Akt) phosphorylation [6,7]. Inhibition of Akt activity ultimately promotes the hyperphosphorylation of tau protein and the production of amyloid β (Aβ) peptides, leading to synaptic dysfunction [8,9]. Moreover, the expression of pro-inflammatory cytokines due to insulin resistance increases the permeability of the blood–brain barrier (BBB) [10,11,12]. It causes neuronal inflammation, neurodegenerative diseases, and cognitive dysfunction in the hippocampal areas [13,14]. Therefore, HFD is associated with type 2 diabetes and leads to Alzheimer’s disease (AD) by increasing diabetic cognitive dysfunction due to insulin resistance [15]. Thus, it is important to evaluate the protective or ameliorating effect of natural resources against HFD-induced diabetic disease. In previous studies, research was conducted to assess the diabetic pathology in an alloxan (ALX) and/or streptozotocin (STZ)-induced animal model without nutritional supplements [16]. However, in contrast to the HFD-induced model, ALX and STZ can cause toxic reactions in other organs and lead to a different aspect of the expression of hyperglycemic status [17]. Therefore, an HFD-induced diabetic model was used for evaluation in this study.

Walnut (*Juglans regia*) is a crop grown worldwide that has various nutrients such as high unsaturated fatty acid, amino acid, mineral contents, fat-soluble vitamins containing α- and γ-tocopherol, and unsaturated fatty acids, such as oleic acid, linoleic acid and linolenic acid [18,19]. In addition, walnuts have various physiologically active compounds such as ellagitannin-based tannins with physiologically bioactive substances [20]. Ellagitannins are polyphenols in which hexahydroxydiphenic acid (HHDP) esters, or their metabolites, are mostly combined with glucose [21]. When ingested into the human body, ellagitannin is hydrolyzed to ellagic acid and/or gallic acid, and those metabolites have physiological activities such as anti-diabetic activity, regulation of hepatic steatosis and serum lipid composition, anti-amnesic effect, and inhibition of inflammasome activation [22,23,24,25]. Based on these bioactive substances, walnut showed antioxidant activity, hyperlipidemia, anti-diabetic activity, and anti-inflammatory activity [18,26,27,28]. Furthermore, walnut showed a protective effect on BBB damage and improved cognitive dysfunction in Aβ-induced mice [29]. However, there are few studies related to the protective effect of walnut against HFD-induced cognitive deficit. Therefore, this study was conducted to evaluate the protective effect of Gimcheon 1ho cultivar walnut (GC) on cerebral disorder by insulin resistance, oxidative stress, and inflammation in HFD-induced diabetic disorder mice.

## 2. Materials and Methods

### 2.1. Chemicals

3-(4,5-dimethylthiazol-2-yl)-2,5-diphenyltetrazolium bromide (MTT), 2′,7′-dichlorodihydrofluorecein diacetate (DCF-DA), fetal bovine serum (FBS), calf serum (CS), dimethyl sulfoxide (DMSO), penicillin, streptomycin, Roswell Park Memorial Institute medium 1640 (RPMI 1640), Dulbecco’s modified Eagle’s medium (DMEM), bovine serum albumin (BSA), hydrogen peroxide (H_2_O_2_) D-glucose, phosphoric acid, thiobarbituric acid (TBA), trichloroacetic acid (TCA), sodium hydroxide, hydroxylamine, hydrogen chloride (HCl), 2,4,6-tris(2-pyridyl)-s-triazine, ferric chloride hexahydrate, sodium acetate, sodium phosphate, sodium azide, fructose, mannitol, sucrose, HEPES sodium salt, digitonin, egtazic acid (EGTA), hydroxylamine, 5,5′-dithiobis-(2-nitrobenzoic acid) (DTNB), tris base, acetic acid, pyruvic acid, malate, 5,5,6,6-tetrachloro-1,1,3,3-tetraethylbenzimidazolylcarbocyanine iodide (JC-1), potassium chloride (KCl), potassium dihydrogen phosphate, magnesium chloride, pyruvate, hydroxylamine, and sodium hydroxide and solvents were obtained from Sigma-Aldrich Chemical Corporation (St. Louis. Mo, USA).

### 2.2. Sample Preparation

The walnut (*Juglans regia*) used in this experiment was obtained from Gimcheon 1ho cultivar (GC) walnut in an experimental forest (production in 2018) of Gimcheon City (Department of Forestry and Greenery) (Gimcheon, Korea) and verified by the National Institute of Forest Science (NIFoS) (Suwon, Korea). According to the previous study, GC presented significant contents of unsaturated fatty acids, total tocopherols, and essential amino acids than other Korean cultivars [30]. The sample was prepared using vacuum lyophilization (Operon, Gimpo, Korea) and stored at −20 °C. The lyophilized sample was extracted with 50-fold 80% ethanol concentrations at 40 °C for 2 h. The extraction sample was filtered through No.2 filter (Whatman Inc, Kent, UK) and concentrated using a vacuum rotary evaporator (N-N series, Eyela Co., Tokyo, Japan). The concentrated extract of GC was lyophilized and stored at −20 °C until use.

### 2.3. UPLC Q-TOF/MS

The main physiological compounds in the GC were analyzed using ultra-performance liquid chromatography–ion mobility separation–quadrupole time of flight/tandem mass spectrometry (UPLC IMS Q-TOF/MS, Vion, Waters Corp., Milford, MA, USA). UPLC separation was investigated with an ACQUITY UPLC BEH C_18_ column (2.1 × 100 mm, 1.7 μm particle size; Waters Corp.). The sample was analyzed using distilled water and acetonitrile (ACN) containing 0.1% formic acid at a flow rate of 0.35 mL/min for 10 min. The mass spectrometer used electrospray ionization (ESI) in the negative ion mode. The capillary and cone voltages were set at 2.5 kV and 40 V, respectively, and the source and desolvation temperatures were performed at 100 °C and 400 °C, respectively. Mass spectral data were collected from m/z 50 to 1500 and processed using MarkerLynx software (Waters Corp.).

### 2.4. In Vitro Cells Study

#### 2.4.1. Cell Culture

PC12 cells with the characteristics of adrenal gland blastoma cell lines were purchased by the Koran Cell Line Bank (Seoul, Korea) and cultured in RPMI 1640 medium with 10% FBS, 50 units/mL penicillin and 100 μg/mL streptomycin. HT22 hippocampal cells were supplied in October 2017 from the Department of Anatomy of the College of Veterinary Medicine, Gyeongsang National University. HT22 cells were cultured in a DMEM medium containing 10% CS, 50 units/mL penicillin and 100 μg/mL streptomycin. Cells were cultured at 37 °C in 5% CO_2_. 

#### 2.4.2. Neuronal Cell Viability and Intracellular ROS

The cell viability was performed using the MTT assay [31]. PC12 and HT22 cells were seeded in 96 well plates (1 × 10^4^ cells/well). After 24 h, the samples and Vitamin C as the positive control were treated. After 24 h, H_2_O_2_ was treated for 3 h. MTT stock solution (10 mg/mL) was added to the pretreated cells for 3 h. The production of violet formazan crystals was determined using a microplate reader (Epoch 2, BioTek, Winooski, VT, USA). The absorbance was measured at 570 nm (determination wavelength) and 655 nm (reference wavelength).

The intracellular ROS contents were performed using the DCF-DA method [31]. After sampling and H_2_O_2_ treatment, a DCF-DA reagent was added to cells and incubated for 50 min. After that, fluorescence levels were performed using a fluorometer (Infinite F200, TECAN, Männedorf, Switzerland) at 485 nm (excitation wavelengths) and 525 nm (emission wavelengths).

### 2.5. Animal Experimental Design

The male C57BL/6 mice (4 weeks old) were obtained from Samtako (Osan, Korea). The experimental animals were randomly assigned to 4 groups: NC group (Normal chow), HFD group, and HFD with sample groups (GC20 and GC50; 20 and 50 mg/kg of body weight, respectively). The mice were selected by body weight and oral glucose tolerance test (OGTT) to confirm the diabetic condition and randomly divided into four groups (*n* = 8; total *n* = 32). After HFD feed was supplied for 12 weeks, samples were orally ingested for 4 weeks to GC20 and GC50 groups. The NC and HFD groups were orally administered with the same amount of water. All animal experiments in the study were in compliance with the Institutional Animal Care and Use Committee (IACUC) of Gyeongsang National University (certificate: GNU-190530-M0028; approved on 30 May 2019).

### 2.6. Glucose Tolerance Test

Fasting blood glucose was measured once a week during sample intake at 15 weeks old. To measure the fasting blood glucose concentration, mice were fasted for 8 h. After 4 weeks, D-glucose (2 g/kg of body weight) was orally administered to all mice at 19 weeks old to evaluate the OGTT. Blood glucose level collected from the tail vein was measured using an Accu-Chek glucose meter at 0, 15, 30, 60, 90, and 120 min (Roche Diagnostics, Basel, Switzerland).

### 2.7. Behavioral Tests

#### 2.7.1. Y-Maze Test

The Y-maze was designed with an internal dimension of 470 mm × 160 mm and 460 mm in height for testing mice. Each mouse was placed at the end of the arm of the maze and allowed to explore freely in the maze for 8 min [32]. The movement and path tracing were recorded using a video system (Smart 3.0, Panlab, Barcelona, Spain).

#### 2.7.2. Passive Avoidance Test

Passive avoidance equipment consists of two compartments as the bright and dark parts, and a door that can pass between them was located between compartments [33]. The compartments were composed of a dark chamber with electrical shock capability. In the first experiment, the mice were located in a bright chamber. When the mice’s four feet entered the dark compartment, a mild foot electrical shock was applied at 0.5 mA for 3 s, and the latency time of the bright compartment was recorded. On the following day, the step-through latency to reenter the dark compartment was measured.

#### 2.7.3. Morris Water Maze (MWM) Test

MWM circular pool designed in diameter of 900 mm × 300 mm height was split into 4 zones as N, S, E, and W by marks on the outside of the pool. In the center of the W quadrant, a platform was submerged below the surface. The pool water was diluted using non-toxic tempera paint. Experimental animals swam to find the platform for a maximum of 1 min and were trained repeatedly for 4 days. After 4 days of training, the platform was removed and retention time in the W zone was measured using a video system (Smart 3.0, Panlab) [34].

### 2.8. Blood Serum Biochemical

After the behavioral tests, mice were fasted for 8 h and sacrificed using exposure to CO_2_. The blood sample was collected at the postcaval vein to evaluate blood serum biochemical analysis. The collected blood samples were centrifuged at 10,000× *g* for 10 min at 4 °C to obtain supernatants of blood. Lactate dehydrogenase (LDH), TG, total cholesterol (TCHO) and high-density lipoprotein cholesterol (HDLC) contents were measured using a clinical chemistry analyzer (Fuji dri-chem 4000i, Fujifilm Co., Tokyo, Japan). Low-density lipoprotein cholesterol (LDLC) level and the ratio of HDLC to TCHO (HTR) were calculated as follows [35].

$$LDLC\;\left(\mathrm{mg}/\mathrm{dL}\right)=TCHO-\left(HDLC+\frac{TG}{5}\right)$$

(1) $$HTR\left(\%\right)=\frac{HDLC}{TCHO}\times 100$$

### 2.9. Preparation of Tissue

Before evaluating the ex vivo test, brain, liver, perirenal white adipose tissue (WAT) fat, retroperitoneal WAT fat, epididymal WAT fat and mesenteric WAT fat tissues were collected, and organ weight was measured. The collected brain tissues were homogenized in a bullet blender (Next Advance Inc., AverillPark, NY, USA). The collected sample was quantitated to calculated using Bradford protein assay [36].

### 2.10. Antioxidant Activity

#### 2.10.1. Malondialdehyde (MDA) Level

To determine MDA level, the supernatant was obtained from brain and liver tissues by centrifugation (5000 rpm, 10 min, 4 °C). The supernatants reacted with 1% phosphoric acid and 0.67% TBA at 95 °C using a water bath for 60 min. The mixtures were spun down at 600× *g* for 10 min and measured at 532 nm (Epoch 2, BioTek) [37]. 

#### 2.10.2. Ferric Reducing Antioxidant Power (FRAP) in Serum

The serum supernatants were obtained as previously described to determine serum antioxidant levels. The serum level of FRAP was measured according to the modified method of Benzie [38]. FRAP solution mixed in 300 mM sodium acetate buffer (pH 3.6), 10 mM TPTZ in 40 mM HCl and 20 mM iron (III) chloride. After 30 min in the dark, the absorbance was measured at 593 nm.

#### 2.10.3. AGEs Formation in Serum

The serum AGEs level was measured according to the method of Sampath [39]. To confirm the serum of AGEs formation, serum was diluted to 1:20 with PBS (pH 7.4). The fluorescence was measured using a fluorometer (Infinite F200, TECAN) at 360 nm (excitation wavelengths) and 460 nm (emission wavelengths) and calculated as a relative unit of controls.

### 2.11. Cerebral Cholinergic System

#### 2.11.1. Acetylcholine (ACh) Level

The ACh level experiment was measured according to the method of Vincent [40]. The supernatant was obtained from brain tissues by centrifugation (12,000× *g*, 30 min, 4 °C). The supernatant was mixed with an alkaline hydroxylamine reagent (2 M hydroxylamine in 0.1 M HCl and 3.5 N NaOH). After mixing, 0.5 N HCl and 0.3 M FeCl_3_ were added and immediately performed using a microplate reader (Epoch 2, BioTek).

#### 2.11.2. Acetylcholinesterase (AChE) Activity

To assess the AChE activity, the supernatant from brain tissues was mixed with 50 mM sodium phosphate buffer and incubated at 37 °C for 15 min [41]. After, the reactants were added to Ellman’s reaction mixture and detected at 405 nm (Epoch 2, BioTek).

### 2.12. Mitochondrial Activity

#### 2.12.1. Extraction of Mitochondria from Brain Tissues

To extract the cerebral mitochondria, brain tissues were homogenized with 10-times as much mitochondrial isolation (MI) buffer (215 mM mannitol, 75 mM sucrose, 0.1% BSA, 20 mM HEPES sodium salt, pH 7.2) with 1 mM EGTA using a bullet blender (Next Advance Inc.). The homogenates were centrifuged at 1300× *g* for 5 min. After, the supernatants of homogenates were centrifuged at 13,000× *g* for 10 min to obtain the pellet. The pellets were mixed with MI buffer containing 0.1% digitonin in DMSO and incubated on ice for 5 min. After, the supernatants were centrifuged at 13,000× *g* for 10 min. Then, the pellets were re-suspended in MI buffer and centrifuged again at 13,000× *g* for 15 min. Finally, the pellets were suspended in MI buffer.

#### 2.12.2. Mitochondrial ROS Contents

Mitochondrial ROS contents were estimated by the DCF-DA assay. The mitochondrial extraction solution was reacted with DCF-DA with respiration buffer (125 mM potassium chloride, 2 mM potassium phosphate, 20 mM HEPES, 1 mM magnesium chloride, and 500 μM EGTA). After incubation for 20 min, the reactant was detected at 535 nm (excitation wavelength) and 458 nm (emission wavelength) using a fluorescence microplate reader (Infinite 200, TECAN) [42].

#### 2.12.3. Mitochondrial Membrane Potential

The level of mitochondrial membrane potential was measured by the JC-1 assay. The mitochondria isolation extract was mixed with JC-1 dye in MI buffer containing 5 mM pyruvate and 5 mM malate and incubated at room temperature for 20 min in the dark. The reactant was detected at 535 nm (excitation wavelength) and 590 nm (emission wavelength) using a fluorescence microplate reader (Infinite 200, TECAN) [42].

### 2.13. Western Blot

The brain tissues were homogenized in lysis buffer (GeneAll Biotechnology, Seoul, Korea) containing 1% protease inhibitor. The homogenates were centrifuged at 13,000× *g* for 10 min at 4 °C to obtain supernatants of homogenates and reacted by loading buffer at 95 °C for 5 min. The protein is separated in electrophoresis through the SDS-PAGE on 8 or 12% SDS gels and transferred to PVDF membranes. The membranes were blocked with 5% skim milk solution in with Tris-buffered saline with 0.5% Tween-20 (TBST), and reacted with primary antibodies at 4 °C. After reaction overnight, membranes were reacted with horseradish peroxidase (HRP)-conjugated secondary antibodies (1:5000) for 1 h. Finally, the membrane reacted with chemiluminescence reagent (TLP-112, TransLab., Daejeon, Korea) was analyzed using the iBright™ CL1000 Imaging System (Thermo Fischer Scientific, Rockford, IL, USA). Strip procedure was conducted using strip buffer (TLP-116.1, TransLab.) at 25 °C for 30 min and re-blotted for evaluation of other protein expressions. β-actin was used as a loading control marker. The density of expression was calculated using ImageJ software (National Institutes of Health, Bethesda, MD, USA). Antibody information is presented in Table 1.

### 2.14. Statistical Analysis

The results for the whole data were proposed as mean ± SD and analyzed by using a one-way analysis of variance (ANOVA) with Duncan’s multiple range test (*p* < 0.05) of SAS 9.4 (SAS Institute Inc., Cary, NC, USA). All experimental data were evaluated for normality and variance homogeneity with the Shapiro–Wilk and Levene’s variance homogeneity tests. Data were statistically represented as significantly different from the NC group (*) and significantly different from the PM group (#), respectively (* and # *p* < 0.05, ** and ## *p* < 0.01).

## 3. Results

### 3.1. UPLC Q-TOF/MS

**Figure 1:**
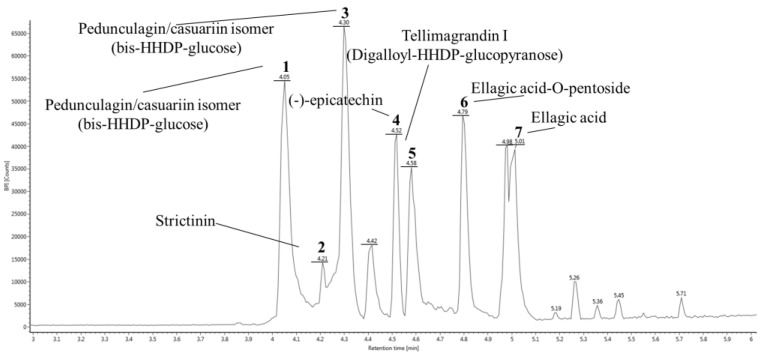
UPLC Q-TOF/MS^E^ chromatography in negative ion mode of ethyl acetate fraction from Gimcheon 1ho (GC) cultivar walnut (*Juglans regia*).

To identify physiological compounds, GC was qualitatively confirmed using UPLC Q-TOF/MS analysis (Figure 1, Table 2). The LC/MS spectrum was compared to that of candidate compounds found in previous reports, especially when compounds in walnut were reported [20,24,27,41]. The ESI-MS^E^ spectra were continuously collected in negative ion mode (M−H)^−^, and the seven main fragments were identified as pedunculagin/casuariin isomer I (bis-HHDP–glucose) (783.06 *m*/*z*), strictinin (633.07 *m*/*z*), pedunculagin/casuariin isomer II (bis-HHDP–glucose) (783.06 *m*/*z*), (-)-epicatechin (289.07 *m*/*z*), tellimagrandin I isomer (digalloyl-HHDP–glucose) (785.07 *m*/*z*), ellagic acid-*O*-pentoside (433.03 *m*/*z*), and ellagic acid (300.99 *m*/*z*) [43].

### 3.2. Protective Effect against H_2_O_2_ and High Glucose-induced Neurotoxicity in PC12 and HT22 Cells

**Figure 2:**
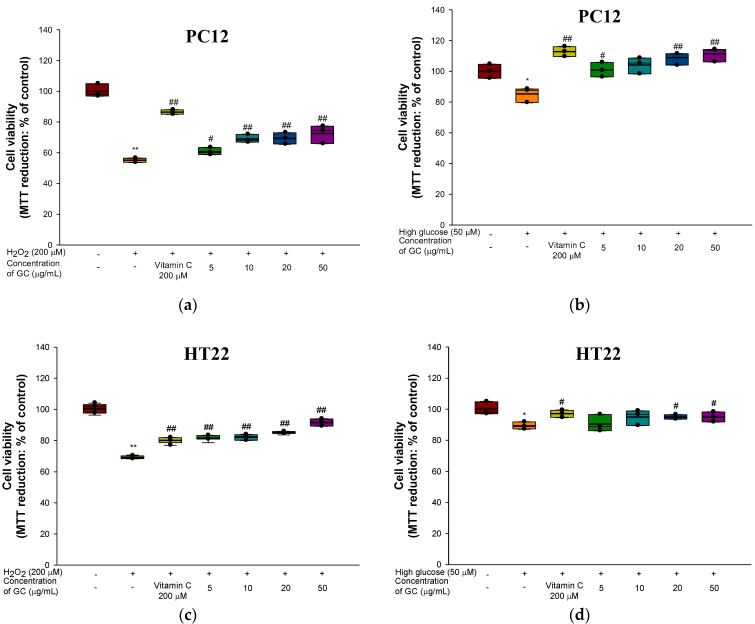
Protective effect of 80% ethanolic extract from Gimcheon 1ho (GC) cultivar walnut (*Juglans regia*): (**a**) cell viability from H_2_O_2_-induced cytotoxicity in PC12 cells; (**b**) cell viability from high-glucose-induced cytotoxicity in PC12 cells; (**c**) cell viability from H_2_O_2_-induced cytotoxicity in HT22 cells; (**d**) cell viability from high glucose-induced cytotoxicity in HT22 cells. Results shown are mean ± SD (*n* = 3). Data are statistically represented with * = significantly different from the NC group, and ^#^ = significantly different from PM group; * and ^#^ *p* < 0.05, ** and ^##^ *p* < 0.01. Bold line indicates mean.

**Figure 3:**
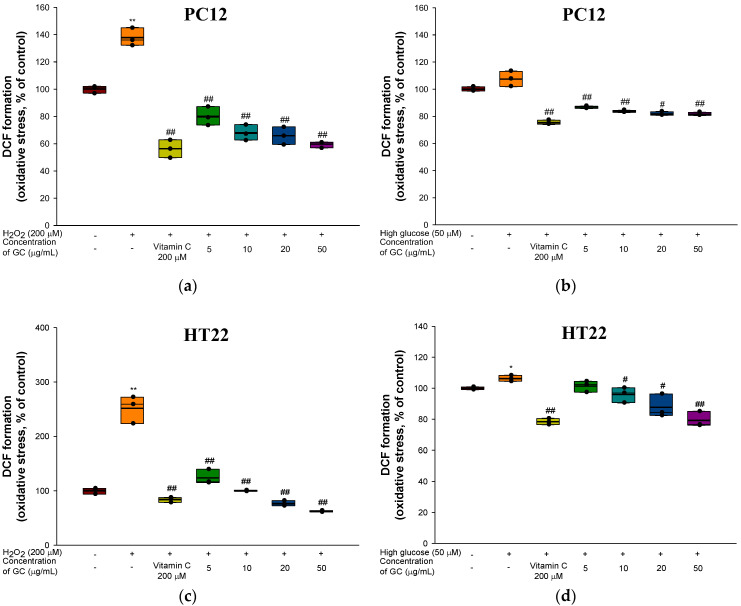
Protective effect of 80% ethanolic extract from Gimcheon 1ho (GC) cultivar walnut (*Juglans regia*): (**a**) ROS production from H_2_O_2_-induced cytotoxicity in PC12 cells; (**b**) ROS production from high glucose-induced cytotoxicity in PC12 cells; (**c**) ROS production from H_2_O_2_-induced cytotoxicity in HT22 cells; (**d**) ROS production from high glucose-induced cytotoxicity in HT22 cells. Results shown are mean ± SD (*n* = 3). Data are statistically represented with * = significantly different from the NC group, and ^#^ = significantly different from PM group; * and ^#^ *p* < 0.05, ** and ^##^ *p* < 0.01. Bold line indicates mean.

The neuroprotective effects of GC against H_2_O_2_ and high glucose in PC12 and HT22 cells are shown in Figure 2 and Figure 3. In the results of cell viability in PC12 cells, the H_2_O_2_-treated group (55.08%) and high glucose-treated group (85.23%) showed cytotoxicity that decreased by 44.92% and 14.77%, respectively, compared to the control group (100%) (Figure 2a,b). On the other hand, treatment of the GC groups with 20 μg/mL (69.32% and 108.89%) and 50 μg/mL (72.50% and 111.33%) showed significantly increased cell viability compared to the H_2_O_2_ and high glucose-treated groups. In addition, the hippocampal HT22 cells were evaluated (Figure 2c,d). Both the 20 μg/mL (84.73% and 91.34%) and 50 μg/mL (94.91% and 94.96%) GC groups showed an increase in cell viability from neurotoxicity caused by H_2_O_2_ (69.64%) compared to the high glucose (89.26%)-treated group. 

Intracellular ROS in the H_2_O_2_ (137.83%) and high-glucose (107.52%)-treated groups increased by 37.83% and 7.52%, respectively, compared to the control group (100%) in PC12 cells (Figure 3a,b). However, the 20 μg/mL (65.88% and 81.78%) and 50 μg/mL (59.67% and 81.60%) GC groups had a decreased level of ROS production. In addition, intracellular ROS production in HT22 increased with H_2_O_2_ (251.46%) and high glucose (106.27%) compared to the control group (100.00%). In contrast, GC treatment showed a remarkable reduction in oxidative stress induced by high glucose at concentrations of 20 μg/mL (75.34% and 87.74%) and 50 μg/mL (61.02% and 79.27%) (Figure 3c,d).

### 3.3. Glucose Tolerance Test

**Figure 4:**
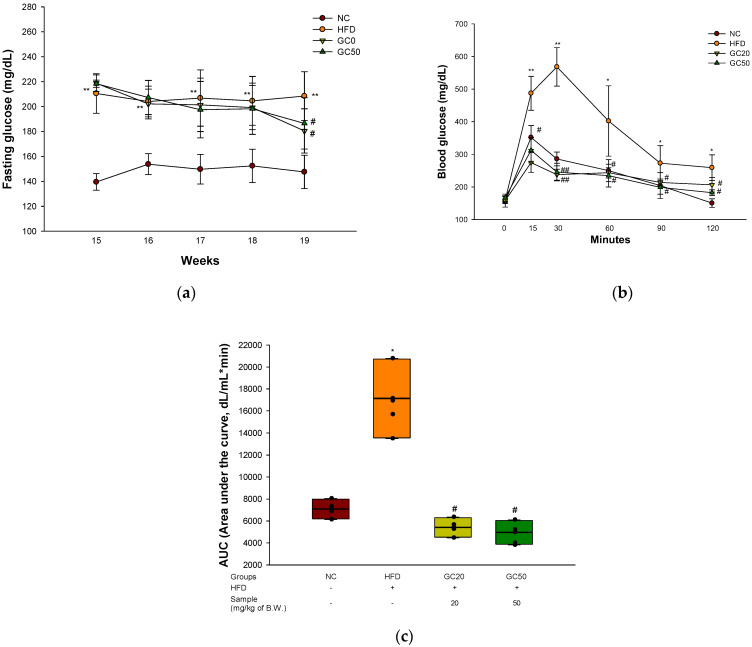
Protective effect of 80% ethanolic extract from Gimcheon 1ho (GC) cultivar walnut (*Juglans regia*) in HFD-induced mice: (**a**) fasting glucose; (**b**) oral glucose tolerance test (OGTT) at 19 weeks old; (**c**) area under the curve (AUC) of OGTT. Results shown are mean ± SD (*n* = 5). Data are statistically represented with * = significantly different from the NC group, and ^#^ = significantly different from PM group; * and ^#^ *p* < 0.05, ** and ^##^ *p* < 0.01. Bold line indicates mean.

To confirm the induction of type 2 diabetes through the HFD, fasting blood glucose was measured at 15 weeks old. The HFD group (210.50 mg/dL) was confirmed to have HFD-induced glucose intolerance, and the fasting glucose level was 1.51 times higher compared to the NC group (139.50 mg/dL) (Figure 4a). The fasting blood glucose level of the HFD group (208.44 mg/dL) significantly increased compared to the NC group (147.57 mg/dL) at 19 weeks old. On the other hand, the GC20 (180.45 mg/dL) and GC50 (186.52 mg/dL) groups showed a significant decrease compared to the HFD group. OGTT was conducted at 0, 15, 30, 60, 90, and 120 min, and the above experimental results were expressed as area under the curve (AUC) (Figure 4b,c). Compared to the normal control group (7090.31 dL/mL∗min), the AUC of the HFD group (17,134.41 dL/mL∗min) showed a 2.42-fold increase, confirming that glucose tolerance increased. However, the GC20 and GC50 groups showed AUC levels of 5413.12 and 4963.79 dL/mL∗min, respectively, and a significantly lower AUC level compared to the HFD group.

### 3.4. Behavioral Tests

**Figure 5:**
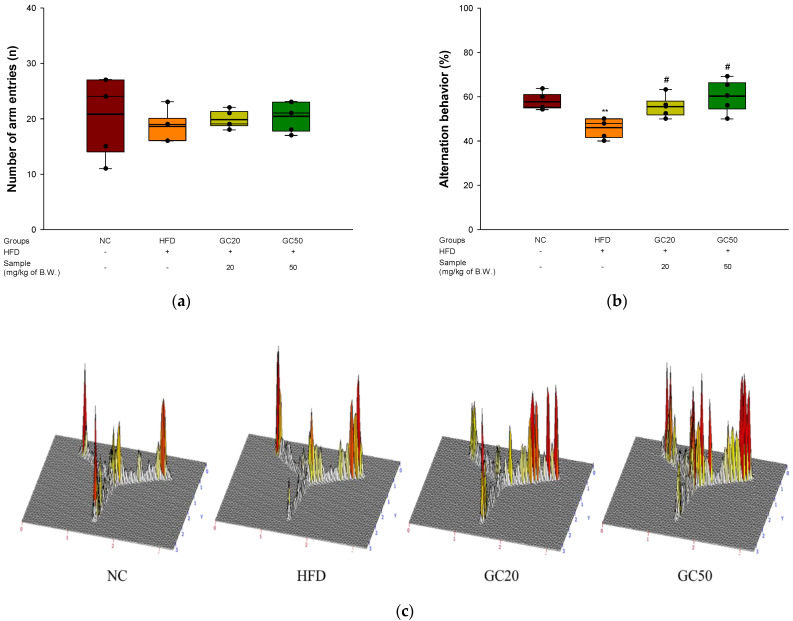

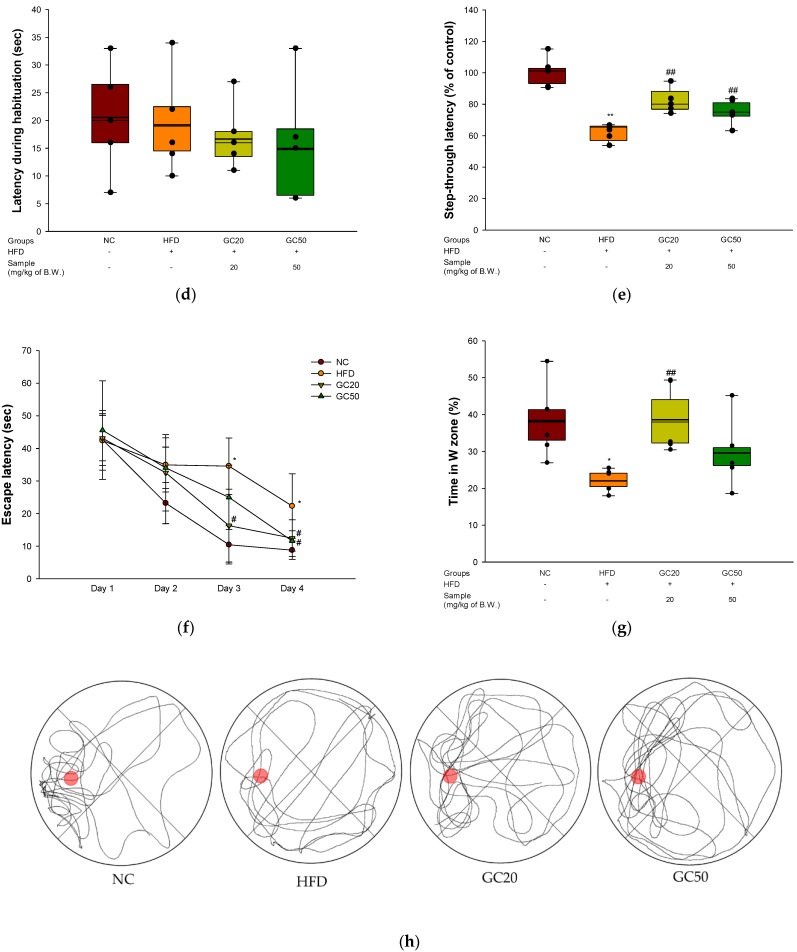
Protective of 80% ethanolic extract from Gimcheon 1ho (GC) cultivar walnut (*Juglans regia*) in HFD-induced mice: (**a**) a number of arm entries; (**b**) spontaneous alternation behavior; (**c**) 3D moving routes in Y-maze test; (**d**) latency during habituation; (**e**) step-through latency in passive avoidance test; (**f**) escape latency in the hidden test; (**g**) retention time in W zone; (**h**) path tracing of each group in Morris water maze (MWM) test. Results shown are mean ± SD (*n* = 5). Data are statistically represented with * = significantly different from the NC group, and ^#^ = significantly different from PM group; * and ^#^ *p* < 0.05, ** and ^##^ *p* < 0.01. Bold line indicates mean.

To investigate spontaneous alternation behavior, a Y-maze test was conducted for 8 min with HFD-induced diabetics [32]. The total number of arm entries (n) in the maze did not show a significant difference between all animals in exercise ability affecting spatial behavior (Figure 5a). The HFD group (45.99%) showed decreased spontaneous alternation behavior compared to that of the NC group (57.63%) (Figure 5b). However, the behavior of the GC groups (GC20, 58.67% and GC50, 62.69%) improved compared to that of the HFD group. In particular, it was confirmed that the above two groups significantly restored ability, similar to the NC group. In an image showing the path tracing of each group of mice as a 3D schematic diagram, the HFD group showed reduced spontaneous alternation behavior. In contrast, the NC and GC groups were similarly indicated in each arm.

A passive avoidance test was performed to measure short-term working memory ability associated with the amygdala [33]. The first step-through latency showed no significant differences between all groups (Figure 5d). There is no prior memory of the average time entering the trial test. In the trial test, the step-through latency of the HFD group (61.80%) was reduced in short-term memory by 38.2% compared with the NC group (100%) (Figure 5e). On the other hand, the GC20 and GC50 groups (82.10% and 75.47%, respectively) were considerably ameliorated compared to the HFD group.

To measure spatial learning acquisition and long-term memory, an MWM test was conducted [34]. In the hidden trial on the fourth day, the escape latency time of the HFD group (22.33 s) was delayed compared to the NC group (8.78 s) (Figure 5f). On the other hand, the GC groups had improved escape latency time following GC administration (GC20, 12.47 s, and GC50, 11.61 s). In the probe test, the retention time in the W zone of the HFD group (22.19%) decreased compared with that of the NC group (38.37%) (Figure 5g). However, the GC groups showed significantly increased retention times (GC20, 38.59% and GC50, 29.71%) compared to the HFD group. In a schematic diagram of the swimming path (Figure 5h), it was seen that the movement in the W zone of the HFD group decreased compared with the NC group. However, the GC groups showed improved movement in the W zone.

### 3.5. Blood Serum Biochemicals and Changes in Weight of Organs

#### 3.5.1. Blood Serum Biochemicals

Serum biomarkers are shown in Table 3. The levels of LDH (782.14 U/L), TG (122.00 mg/dL), TCHO (215.57 mg/dL), and LDLC (59.74 mg/dL) of the HFD group increased higher than those of the NC group (LDH, 280.29 U/L; TG, 95.00 mg/dL; TCHO, 124.14 mg/dL; LDLC, 22.00 mg/dL). The GC groups showed no significantly decreased TG (GC20, 118.14 mg/dL) and TCHO (GC20, 215.00 mg/dL, GC50 201.29 mg/dL) compared to the HFD group. However, the levels of LDH (660.43 U/L and 537.57 U/L, respectively) and LDLC (39.53 mg/dL and 33.33 mg/dL, respectively) in the GC groups seemed to significantly decrease compared to the HFD group. In particular, the GC50 group had reduced LDH (537.57 U/L) and TG (109.29 mg/dL) levels compared to the HFD group. In addition, the HFD groups increased HDLC (131.43 mg/dL) and decreased HTR (60.77%) compared to the NC group (83.86 mg/dL, 67.63%, respectively). On the other hand, the GC groups had improved HDLC (157.43 mg/dL, 150.86 mg/dL, respectively) and HTR (70.80% and 69.61%, respectively) compared to the HFD group.

#### 3.5.2. Changes in Weight of Organs

To estimate fat accumulation, organs mass is presented in Table 4. The weight of the brain showed no significant differences between all groups. However, the weight of the liver (2.67 g), hepatic lipid (19.58 mg/g), perirenal WAT fat (0.35 g), retroperitoneal WAT fat (1.23 g), epididymal WAT fat (2.22 g), mesenteric WAT fat (0.90 g) and total WAT fat (4.39 g) in the HFD group increased compared to the NC group (liver, 1.28 g; hepatic lipid, 6.83 mg/g; perirenal WAT fat, 0.12 g; retroperitoneal WAT fat, 0.29 g; epididymal WAT fat, 1.25 g; mesenteric WAT fat, 0.25 g and total WAT fat, 2.00 g). The liver weight and hepatic lipid in the GC20 (1.70 g, 15.28 mg/g, respectively) and GC50 (1.54 g, 10.28 mg/g, respectively) groups were reduced compared to the HFD group. In addition, the perirenal (GC20, 0.40 g and GC50, 0.40 g), retroperitoneal (GC20, 0.71 g and GC50, 0.75 g), and epididymal (GC20, 1.65 g and GC50, 0.94 g) WAT fat and total WAT fat (GC20, 3.50 g and GC50, 3.26 g) of the GC groups were suppressed compared to the HFD group. However, mesenteric WAT fat showed no significant difference between the GC groups (GC20, 0.91 g and GC50, 0.95 g) compared to the HFD group.

### 3.6. Inhibition of Lipid Peroxidation

**Figure 6:**
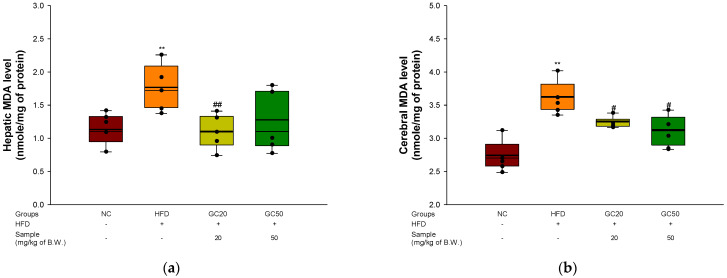
Protective effect of 80% ethanolic extract from Gimcheon 1ho (GC) cultivar walnut (*Juglans regia*) on malondialdehyde (MDA) contents in HFD-induced mice biochemical changes related with antioxidant system: (**a**) hepatic MDA level; (**b**) cerebral MDA level Results shown are mean ± SD (*n* = 5). Data are statistically represented with * = significantly different from the NC group, and ^#^ = significantly different from PM group; ^#^ *p* < 0.05, ** and ^##^ *p* < 0.01. Bold line indicates mean.

To estimate the inhibition of lipid peroxidation in hepatic and cerebral tissues, the hepatic and cerebral levels of MDA were assessed. The MDA level in the hepatic and cerebral tissues of the HFD group (1.73 and 3.57 mmole/mg of protein, respectively) increased compared to the NC group (1.09 and 2.69 mmole/mg of protein, respectively) (Figure 6a,b). Conversely, the levels in the GC20 (1.09 and 3.20 mmole/mg of protein) and GC50 (1.20 and 3.04 mmole/mg of protein) groups decreased more than the HFD group in hepatic and cerebral tissues, respectively.

### 3.7. Serum Level of FRAP and AGEs Formation 

**Figure 7:**
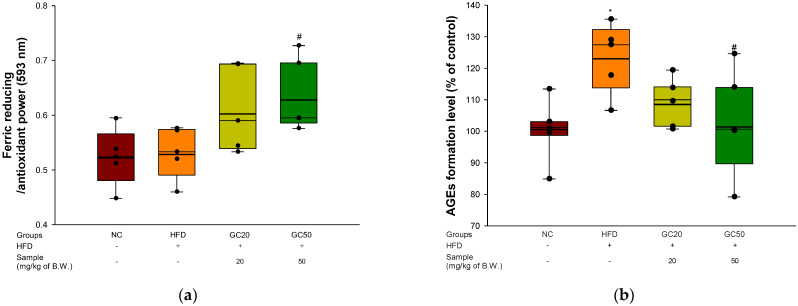
Protective of 80% ethanolic extract from Gimcheon 1ho (GC) cultivar walnut (*Juglans regia*) in HFD-induced mice: (**a**) serum level of FRAP; (**b**) serum level of AGEs formation. Results shown are mean ± SD (*n* = 5). Data are statistically represented with * = significantly different from the NC group, and ^#^ = significantly different from PM group; * and ^#^ *p* < 0.05. Bold line indicates mean.

To examine the serum level of FRAP and AGEs formation in HFD-induced mice, a FRAP and AGEs formation assay was performed. There was no difference in FRAP levels in serum between the NC group (0.52) and the HFD group (0.53) (Figure 7a). However, the GC groups (GC20, 0.61 and GC50, 0.63) were increased. AGEs formation in serum was evaluated. The HFD group (121.02%) had accumulated AGEs compared to the NC group (100%) (Figure 7b). Conversely, the GC groups (GC20, 110% and GC50, 105%) inhibited AGEs formation in serum.

### 3.8. Cholinergic System

**Figure 8:**
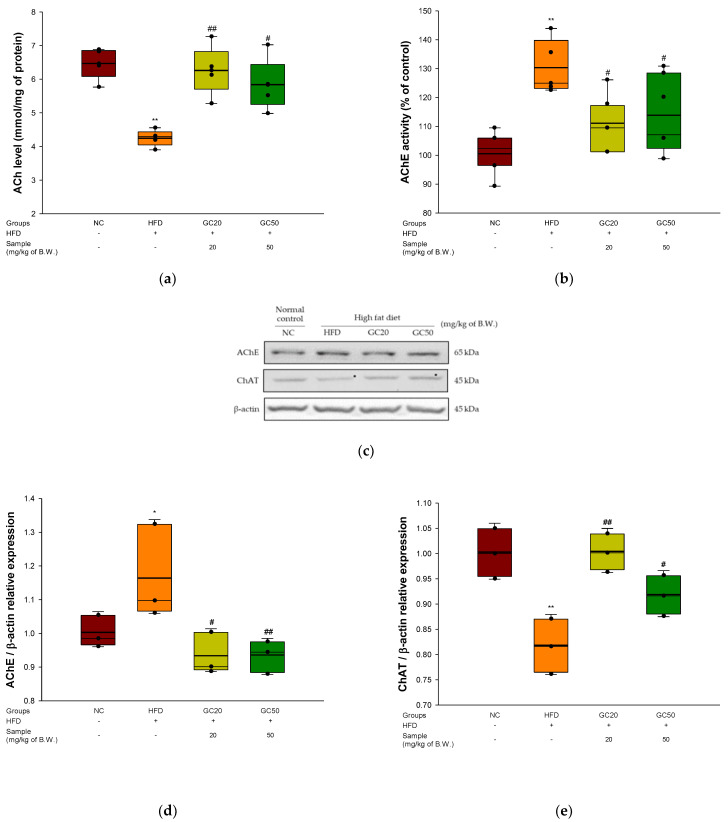
Protective effect of 80% ethanolic extract from Gimcheon 1ho (GC) cultivar walnut (*Juglans regia*) on HFD-induced mice: (**a**) ACh level; (**b**) AChE activity; (**c**) representative western blots for total protein and expression of AChE, ChAT and β-actin; (**d**) protein expression levels of AChE; (**e**) protein expression levels of ChAT. Results shown are mean ± SD (**a**,**b**: *n* = 5, **c**–**e**: *n* = 3). Data are statistically represented with * = significantly different from the NC group, and ^#^ = significantly different from PM group; * and ^#^ *p* < 0.05, ** and ^##^ *p* < 0.01. Bold line indicates mean.

To assess the cognitive functions of the cholinergic system in cerebral tissue, ACh levels, AChE activity, and expression levels of AChE and choline acetyltransferase (ChAT) were evaluated. The ACh levels of the HFD group (4.30 mmol/mg of protein) decreased compared to the NC group (6.47 mmol/mg of protein) (Figure 8a). However, the ingestion of GC showed considerably increased ACh levels (6.26 mmol/mg of protein and 5.84 mmol/mg of protein, respectively). The AChE activity of the HFD group (130.27%) increased compared to the NC group (100%) (Figure 8b). However, the GC groups (GC20, 112.07% and GC50, 115.24%) reduced AChE activity compared to the HFD group. 

The expression of AChE and ChAT is shown in Figure 8c–e. The AChE expression level in the HFD group (1.16) was up-regulated compared to the NC group (1.00). On the other hand, the GC groups (GC20, 0.93 and GC50, 0.93) suppressed AChE expression compared to the HFD group. The ChAT expression level in the HFD group (0.81) was down-regulated compared to the NC group (1.00) (Figure 8e). However, the GC groups (GC20, 1.00 and GC50, 0.92) showed up-regulated ChAT expression compared to the HFD group.

### 3.9. Mitochondrial Activity

**Figure 9:**
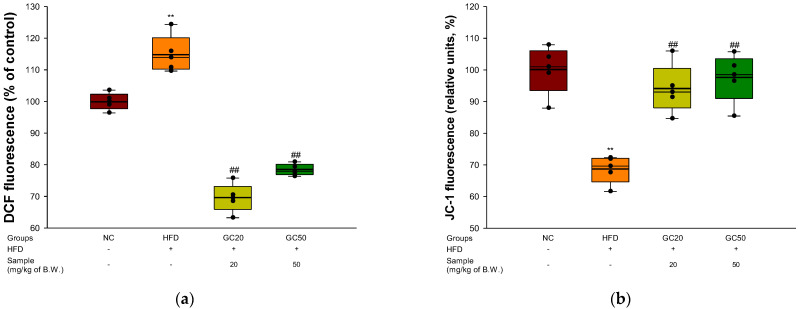
Protective effect of 80% ethanolic extract from Gimcheon 1ho (GC) cultivar walnut (*Juglans regia*) on mitochondrial dysfunction in HFD-induced mice: (**a**) cerebral ROS contents; (**b**) cerebral MMP levels Results shown are mean ± SD (*n* = 5). Data are statistically represented with * = significantly different from the NC group, and ^#^ = significantly different from PM group; ** and ^##^ *p* < 0.01. Bold line indicates mean.

To substantiate mitochondrial function in cerebral tissues, ROS production and MMP levels were evaluated. The ROS production of the HFD group in cerebral tissues (114.93%) increased compared to the NC group (100%) (Figure 9a). However, the GC20 (69.53%) and GC50 (78.40%) groups showed considerably decreased ROS production compared with the HFD group. The MMP of the HFD group in cerebral tissues (68.61%) decreased compared to the NC group (100%) (Figure 9b). In contrast, the GC20 (94.88%) and GC50 (97.50%) groups showed restored MMP in cerebral tissues compared to the HFD group. 

### 3.10. Protein Expression in Cerebral Tissue

#### 3.10.1. Synaptic Disorders and Neuronal Apoptosis

**Figure 10:**
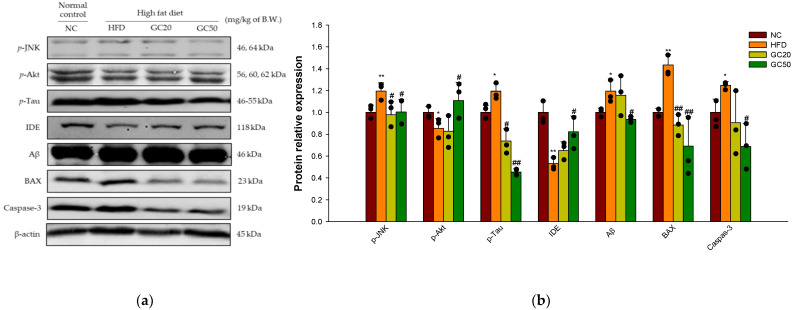
Protective effect of 80% ethanolic extract from Gimcheon 1ho (GC) cultivar walnut (*Juglans regia*) on HFD-induced synaptic disorders and neuronal apoptosis in mice brain tissues: (**a**) representative Western blots for total protein and expression; (**b**) protein expression levels of *p*-JNK, *p*-Akt, *p*-tau, IDE, Aβ, BAX, caspase-3. Results shown are mean ± SD (*n* = 3). Data are statistically represented with * = significantly different from the NC group, and ^#^ = significantly different from PM group; * and ^#^ *p* < 0.05, ** and ^##^ *p* < 0.01. Bold line indicates mean.

Cerebral protein expressions associated with synaptic disorders and neuronal apoptosis are shown in Figure 10a. *p*-JNK (1.20), *p*-tau (1.19), and Aβ (1.20) expression levels in the HFD group significantly increased compared to the NC group (Figure 10b). For the GC20 and GC50 groups *p*-JNK (0.98 and 1.00, respectively), *p*-tau (0.74 and 0.45, respectively), and Aβ (1.16 and 0.94, respectively) expression levels were significantly decreased. In addition, the HFD group showed a decreased expression level of *p*-Akt (Ser 473) (0.85) and insulin-degrading enzyme (IDE) (0.53) compared to the NC group (1.00) (Figure 10b). However, the GC20 and GC50 groups statistically up-regulated *p*-Akt (Ser 473) (0.82 and 1.11, respectively) and IDE (0.65 and 0.82, respectively) expression levels compared to the HFD group. Furthermore, the expression levels of B-cell lymphoma (BCl)-2 associated X protein (BAX) (1.43) and caspase-3 (1.25) were significantly up-regulated in the HFD group compared to the NC group (1.00) (Figure 10b). In contrast, the GC20 and GC50 groups had significantly down-regulated BAX (0.88 and 0.69, respectively) and caspase-3 (0.91 and 0.69, respectively) expression levels compared to the HFD group.

#### 3.10.2. Neuroinflammation

**Figure 11:**
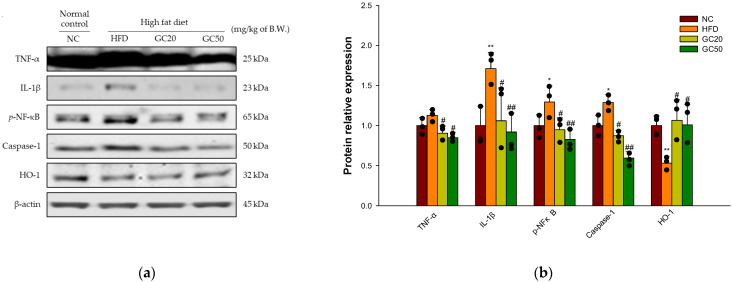
Protective effect of 80% ethanolic extract from Gimcheon 1ho (GC) cultivar walnut (*Juglans regia*) on HFD-induced neuroinflammation in mice brain tissues: (**a**) representative Western blots for total protein and expression; (**b**) protein expression levels of TNF-α, IL-1β, *p*-NFκB, caspase-1, and HO-1. Results shown are mean ± SD (*n* = 3). Data are statistically represented with * = significantly different from the NC group, and ^#^ = significantly different from PM group; * and ^#^ *p* < 0.05, ** and ^##^ *p* < 0.01. Bold line indicates mean.

Cerebral protein expressions related to neuronal inflammation are shown in Figure 11a. The expression levels of inflammation-related factors TNF-α (1.13), IL-1β (2.11), *p*-NFκB (1.29) and caspase-1 (1.29) were significantly increased in the HFD group compared to the NC group (1.00) (Figure 11b). On the other hand, the GC20 and GC50 groups statistically ameliorated TNF-α (0.90 and 0.85, respectively), IL-1β (1.30 and 1.14, respectively), *p*-NFκB (0.95 and 0.83, respectively) and caspase-1 (0.87 and 0.60, respectively) expression levels compared to the HFD group. Moreover, heme oxygenase-1 (HO-1) expression levels in the HFD group (0.53) were significantly down-regulated compared to the NC group (1.00) (Figure 11b). HO-1 (1.07 and 1.01) expression levels were significantly up-regulated in the GC20 and GC50 groups compared to the HFD group.

## 4. Discussion

Diabetes is a metabolic disease caused by inflammation in a complex immunological process and is related to HFD intake, leading to obesity [3,44]. In general, HFD-induced systemic oxidative stress is associated with reduced insulin sensitivity and the promotion of inflammation following damage in a variety of organs [45]. Insulin resistance and inflammation are the major factors contributing to the degree of severity of the progression of cognitive impairment [10]. Therefore, this study was conducted to evaluate the protective effect of GC on increased oxidative stress, inflammation, and insulin resistance in HFD-induced diabetic disorder.

Type 2 diabetes is characterized by hyperglycemia related to impaired glucose tolerance [46]. High glucose causes reactive oxygen and nitrogen species, primarily mitochondrial dysfunction [47]. Therefore, the abnormal metabolism of glucose, such as hyperglycemia, leads to excess free radical and oxidative stress generation [48]. Chronic hyperglycemia is considered a cause of brain injury by increasing neuroinflammation and apoptotic neuronal death [49]. This study showed that GC effectively protected neuronal cells against H_2_O_2_ and high glucose-induced neurotoxicity and reduced ROS production in PC12 and HT22 cells. Walnut, which has an abundance of polyunsaturated fatty acids (PUFAs) and polyphenolic compounds, reduced cerebral oxidative stress and inflammatory reaction by enhancing neuronal signaling [30,50]. According to Muthaiyah et al., walnut extract protected neuronal cells against Aβ-mediated cytotoxicity by increasing the capacity of endogenous antioxidant defenses and modulating the cellular redox state [51]. Therefore, GC might be effective in ameliorating ROS production and neurodegeneration.

Increased levels of circulating FFAs are implicated in pancreatic β-cell dysfunction, leading to glucose intolerance [15]. High levels of serum FFA are an important cause of obesity-associated insulin resistance and complications of dyslipidemia [52]. HFD caused dyslipidemic changes by increasing serum levels such as triacylglycerol, TCHO, LDLC, and very-low-density lipoprotein (VLDLC) and decreasing HDLC levels [53]. Dyslipidemia increases oxidative stress through a lipid chain reaction related to endothelial dysfunction and inflammation [45]. Increased oxidative stress induced by insulin resistance, dyslipidemia, and impaired glucose tolerance ultimately lead to type 2 diabetes [54,55]. On the other hand, GC intake improved glucose tolerance and aberrant lipid profiles in serum and significantly increased HDLC content. According to previous studies, the administration of walnut extract for 6 weeks in streptozotocin (STZ)-induced diabetic CD rats reduced blood glucose levels and increased insulin sensitivity by ameliorating insulin resistance [56]. Walnut oil-derived PUFA administration decreased fasting blood glucose and increased hepatic glycogen levels in pregnant diabetic rats [57]. In addition, walnut significantly reduced serum cholesterol, LDLC, TG, and VLDLC levels and increased HDLC compared to STZ-induced diabetic rats [58]. Moreover, the linoleic acid and α-linolenic acid in walnut significantly lowered TCHO, LDL, and TG in obese females [58]. According to Shi et al., the administration of walnut polyphenol extracts inhibited intestinal lipid absorption in HFD-induced mice [26]. Tellimagrandin I, one of the walnut polyphenols, inhibited the TG mechanism in the hyperglycemia model [43]. Consequently, GC consumption plays an important role in improving glucose tolerance and serum lipid levels and preventing type 2 diabetes.

HFD contributes to chronic inflammation and hyperglycemia, resulting in increased oxidative stress [44]. Exposure to hyperglycemia conditions results in irreversible AGEs by rearrangement without degradation of reversible Amadori-type initial glycation products [59]. The accumulation of AGEs induces oxidative stress and contributes to insulin resistance and tissue damage [60]. High levels of oxidative stress destroy cellular membranes, resulting in the overproduction of cytotoxic aldehyde byproducts such as MDA [10]. The production of lipid peroxidation can react with cellular proteins or DNA to form adducts, causing biomolecular damage [37]. In addition, oxidative stress increases the permeability of the BBB, alters the morphology of the brain, damages the central nervous system (CNS), and ultimately promotes neurodegenerative disorders [61]. This study showed that GC significantly reduced AGEs and inhibited MDA in the brain. Furthermore, GC with significantly increased FRAP ability in serum shows antioxidant activity in HFD. According to previous studies, intake of walnut in mice increased plasma antioxidant capacity [62]. Walnut prevented oxidative damage in tissues by reducing lipid oxidation in an ethanol-induced rat model [63]. In addition, it was reported that the unsaturated fatty acids in walnuts effectively inhibit lipid peroxidation [21,62,64]. In conclusion, GC inhibits hepatic and cerebral lipid peroxidation and the formation of serum AGEs and increases serum antioxidant activity to improve HFD-induced oxidative stress.

High levels of FFA are oxidized by β-oxidation in liver mitochondria or accumulated to TG by esterification [65]. The production of electron donors such as reduced nicotinamide adenine dinucleotide (NADH) and dihydroflavine-adenine dinucleotide (FADH_2_) by β-oxidation produces ATP according to the mitochondrial electron transport chain (ETC) [47]. However, increased β-oxidation of FFA results in significant loss of ETC due to overproduction of ROS in mitochondria [66]. Abnormally damaged ETC activity reduces mitochondrial membrane potential and ATP synthesis [67]. Brain mitochondrial dysfunction occurring in association with neuronal insulin resistance could lead to the development of neuronal damage [10]. However, the administration of GC improved mitochondrial membrane potential function and suppressed oxidative stress in the brain. Similar to this, ellagitannin of walnut polyphenol effectively improved hyperlipidemia and metabolic syndrome by enhancing peroxisomal β-oxidation [42]. In addition, walnut showed mitochondrial ROS scavenging activity and improvement of neuronal energy metabolism in Aβ-injected mice [29]. Therefore, GC may protect against neuronal loss by improving the function of cerebral mitochondria.

HFD-induced diabetes is a risk factor for cognitive impairment in AD [68]. Hippocampus, amygdala, and cerebellar granule cells are susceptible to oxidative stress, and oxidative stress-induced brain damage significantly affects behavioral and cognitive decline [60]. Increased oxidative stress derived from HFD causes neuronal inflammation in the hippocampal region and impairs hippocampal synaptic plasticity, learning, and memory [14]. When spatial memory in a rodent model is assessed, hippocampal-dependent memory/learning deficits are highly correlated with cognitive–behavioral impairments [69]. In this study, the HFD group showed significant memory impairment in behavioral tests [27,70]. On the other hand, the administration of GC showed improvement in spatial learning and memory function in HFD-induced mice. According to previous studies, walnut could improve the memory and cognition of d-galactose-induced aging mice in behavioral tests [70]. In addition, walnut extract has been shown to ameliorate behavioral disorders and memory deficits in an Aβ_1-42_-induced mouse model [29]. Similar to previous studies, it is suggested that GC reduces memory deficits in HFD-induced behavioral disorders.

Cholinergic neural circuits play essential roles in memory dysfunction [41]. However, alterations of cholinergic function are associated with memory impairment in animals [71]. Ach, a cholinergic neurotransmitter, is released from a broad range of cortical and subcortical sites at the end of synapses and plays an essential role in cognitive functions such as learning and memory [72]. Normally, ACh is synthesized by ChAT by mediating the transfer of the acetyl group from acetyl CoA to choline at the synaptic endings of cholinergic neurons [73]. However, excessive intake of HFD results in cholinergic dysfunction such as abnormal changes in ChAT and AChE expression in many brain regions, including the hypothalamus, hippocampus, amygdala, and cortex [74]. In this study, GC intake restored the cholinergic system in brain tissue and significantly improved the protein expression levels of ChAT and AChE. According to previous studies, walnut inhibited cerebral AChE activity, indicating improvement in learning and memory in D-galactose-induced aging mice [71]. In addition, ellagitannins such as tellimagradin I purified from *Trapa taiwanensis* Nakai hulls showed AChE inhibitory activities in scopolamine-induced amnesia mice [75]. In conclusion, GC containing various ellagitannins has an effect on the cholinergic system by protecting against cognitive dysfunction in HFD-induced mice.

Excessive intake of HFD causes various damages to brain tissue, which is similar to the pathology of AD [9]. HFD impairs JNK/Akt signaling related to brain insulin resistance and leads to cognitive dysfunction [10]. Inhibited Akt activity increases the phosphorylation of GSK-3β and continuously induces hyperphosphorylation of tau protein and aggregation of neurofibrillary tangles (NFTs), leading to synaptic dysfunction and apoptosis [8]. Another pathological feature of HFD-induced insulin resistance is the presence of islet amyloid deposits by the Aβ_1-40_ and Aβ_1-42_ peptides due to increased γ-secretase activity in the brain [5]. IDE is one of the Aβ-degrading enzymes involved in the selective cleavage of Aβ peptides in combination with insulin degradation and prevents the formation of amyloid deposits [76]. However, when insulin is increased in the blood, IDE does not cleavage Aβ effectively and causes Aβ neurotoxicity, which eventually leads to AD amyloidosis [9,77]. Neurotoxicity via the accumulation of Aβ peptides and tau NFTs down-regulates BCl-2 at synapses and dendrites and up-regulates BAX to activate downstream signals of apoptosis [78]. Activation of BAX at the mitochondrial surface forms macropores on the mitochondrial outer membrane, leading to the release of cytochrome c into the cytosol [79]. In addition, the release of cytochrome c from mitochondria induces caspase-3 activation, which stimulates signaling pathways and leads to synaptic loss and neuronal apoptosis [80]. In the present study, GC caused a significantly improved HFD-induced JNK/Akt signaling pathway and suppressed the expression of tau and Aβ. In addition, GC inhibited BAX and caspase-3 activation and protected against HFD-induced cerebral apoptosis. According to Ma et al., walnut supplementation significantly reduced the activated cell death-associated expression of *p*-p38 mitogen-activated protein kinase (p38K) and *p*-JNK [62]. In addition, it was confirmed that ingestion of walnut extract inhibited Aβ and tau production in brain tissue by improving the Akt signaling pathway in Aβ-induced mice [29]. Additionally, ellagic acid dose-dependently decreased pathogenic Aβ oligomers and Aβ cytotoxicity in origin SH-SY5Y cells [81]. Moreover, walnut protected against apoptosis by reducing BAX protein levels and cytochrome c release in UVB-induced origin HaCaT cells [82]. Furthermore, walnut peptides inhibited apoptosis by inhibiting the expression of cytochrome c and caspase-3 [83]. These results suggest that GC regulates JNK/Akt signaling to protect against significantly suppressed insulin resistance-mediated synaptic disorders and apoptosis.

HFD-mediated excessive FFA activates inflammation by releasing inflammatory cytokines in brain tissue [10,84]. Increased expression of various pro-inflammatory cytokines such as TNF-α and IL-1β activates NFκB, a transcriptional activator [62]. The active NFκB promotes the transcription of NFκB-dependent genes, such as leucine-rich repeat (NLR) pyrin domain containing 3 (NLRP3), pro-IL-1β, and TNF- α in the nucleus [85]. The NLRP3 family member forms, triggering autocatalytic activation of caspase-1 and suppresses nuclear factor erythroid 2–related factor 2 (Nrf2) expression [86]. This reaction reduces the expression of HO-1 with a strong antioxidant effect and stimulates the production of IL-1β and TNF-α [87,88]. Thus, excessive inflammatory cytokine expression contributes to the pathogenesis of inflammatory responses and cognitive impairment [11,89]. GC restored neuroinflammation by suppressing protein expression levels of TNF-α, IL-1β, *p*-NFκB, caspase-1, and HO-1. In a previous study, walnut peptides inhibited NFκB pathway activation and attenuated the neurotoxic cascade by overexpression of IL-1β and TNF-α [83]. In addition, walnut reduced the production of TNF-α, IL-1β, and IL-6 by suppressing their mRNA expressions in LPS-induced mice [89]. Furthermore, walnut-derived peptides protected insulin resistance and decreased oxidative stress by activating HO-1 in high glucose-induced origin HepG2 cells [27]. Ellagic acid protected against inflammation such as nitric oxide (NO), MDA, IL-1β, TNF-α, cyclooxygenase 2 (COX-2), and NFκB expression in carrageenan-induced rats [90]. Therefore, GC may improve cognitive function by ameliorating neurodegenerative disorders by reducing neuroinflammation.

**Figure 12:**
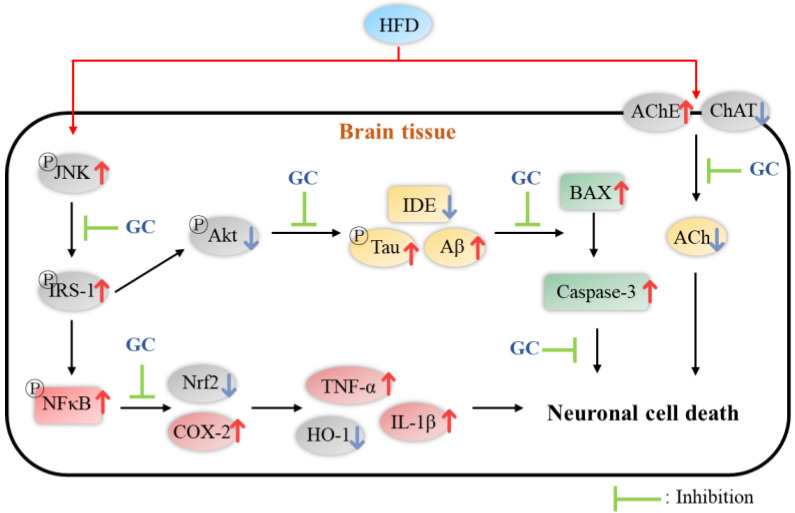
A schematic illustration shows the immunomodulatory effect of 80% ethanolic extract from Gimcheon 1ho (GC) cultivar walnut (*Juglans regia*) on HFD-induced neuroinflammation in mice brain tissues.

Overall, walnut showed a protective effect against HFD-induced diabetic symptoms such as glucose tolerance, diabetic cognitive dysfunction, hyperlipidemia, mitochondrial deficit, apoptosis, and neuronal inflammation. Walnut contains various physiological compounds such as PUFA, α-linolenic acid, ellagitannins, ellagic acid, and bioactive peptides. According to previous studies, walnut-derived PUFA decreased fasting blood glucose, TCHO, LDL, and TG levels and inhibited lipid peroxidation [21,57,58,62]. Walnut ellagitannin, such as tellimagradin I and ellagic acid, enhanced peroxisomal β-oxidation and reduced the TG mechanism and inflammatory responses [42,90]. In addition, ellagitannins protected against synaptic dysfunction by regulating AChE activity and pathogenic Aβ oligomers [75,81]. Various bioactive peptides of walnut inhibited apoptosis by regulating mitochondrial apoptosis and suppressed insulin resistance by increasing the HO-1 pathway [27,83]. In conclusion, based on these physiological activities, GC showed protective effects against HFD-induced diabetic dysfunctions through complex and diverse pathways (Figure 12).

## 5. Conclusions

In summary, based on this study, GC showed a significant neuroprotective effect in neuronal cells and hippocampal cells induced by H_2_O_2_ and high glucose stresses. GC restored behavioral dysfunction in HFD-induced C57BL/6 mice. In addition, GC protected antioxidant and cholinergic systems and improved mitochondrial dysfunction. Furthermore, the administration of GC suppressed synaptic disorders by regulating AChE and ChAT expression. In addition, GC inhibited cerebral cytotoxicity via JNK cascade signaling. GC down-regulated inflammatory responses by regulating the protein expression of TNF-α, IL-1β, *p*-NFκB, caspase-1, and HO-1. In conclusion, it is suggested that GC extract can be used as a material for functional foods that improve memory loss and cognitive dysfunction by regulating synaptic function and inflammation.

## Figures and Tables

**Table 1:** List of primary antibody information used in this study.

Antibody	Catalog	Conc.	Manufacturer
β-actin	sc-69879	1:1000	Santa Cruz Biotech (Dallas, TX, USA)
AChE	sc-373901	1:1000	Santa Cruz Biotech (Dallas, TX, USA)
*p*-JNK	sc-6254	1:1000	Santa Cruz Biotech (Dallas, TX, USA)
*p*-Akt	sc-514032	1:1000	Santa Cruz Biotech (Dallas, TX, USA)
*p*-tau	sc-12952	1:1000	Santa Cruz Biotech (Dallas, TX, USA)
IDE	sc-393887	1:1000	Santa Cruz Biotech (Dallas, TX, USA)
Aβ	sc-28365	1:1000	Santa Cruz Biotech (Dallas, TX, USA)
BAX	sc-7480	1:1000	Santa Cruz Biotech (Dallas, TX, USA)
Caspase-1	sc-392736	1:1000	Santa Cruz Biotech (Dallas, TX, USA)
TNF-α	sc-393887	1:1000	Santa Cruz Biotech (Dallas, TX, USA)
IL-1β	sc-4592	1:1000	Santa Cruz Biotech (Dallas, TX, USA)
HO-1	sc-136960	1:1000	Santa Cruz Biotech (Dallas, TX, USA)
ChAT	20747-1AP	1:1000	Bioneer (Daejeon, Korea)
*p*-NF-κB	#3033	1:1000	Cell Signaling Tech (Danvers, MA, USA)
Caspase-3	CSB-PA05689A0Rb	1:1000	Cusabio (Hubei, China)

**Table 2:** Identification of main compounds of Gimcheon 1ho (GC) cultivar walnut (*Juglans regia*).

Peak No.	RT ^a^ (min)	Negative Ion Mode (*m*/*z*)	MS^E^ Fragments (*m*/*z*)	Proposed Compound	Compounds Formula
1	4.05	783.06	481.06, 300.99, 275.02	Pedunculagin/casuariin isomer (bis-HHDP-glucose) I	C_34_H_24_O_22_
2	4.21	633.07	481.06, 300.99	Strictinin	C_27_H_22_O_18_
3	4.30	783.06	481.06, 300.99, 275.02	Pedunculagin/casuariin isomer (bis-HHDP-glucose) II	C_34_H_24_O_22_
4	4.52	289.07	245.08	(−)-Epicatechin	C_15_H_14_O_6_
5	4.58	785.07	483.07, 300.99, 275.02, 169.01	Tellimagrandin I (Digalloyl-HHDP-glucopyranose)	C_34_H_26_O_22_
6	4.79	433.03	300.99, 299.98	Ellagic acid-O-pentoside	C_19_H_14_O_12_
7	5.01	300.99	302.00	Ellagic acid	C_14_H_6_O_8_

^a^ RT means retention time. All results were detected in negative ion mode using UPLC Q-TOF/MS^E^.

**Table 3:** Effect of 80% ethanolic extract from Gimcheon 1ho (GC) cultivar walnut (*Juglans regia*) on serum biomarkers.

Groups	NC	HFD	GC20	GC50
LDH (U/L)	280.29 ± 79.82	782.14 ± 231.88 **	660.43 ± 244.87	537.57 ± 127.19 ^#^
TG (mg/dL)	95.00 ± 19.17	122.00 ± 11.49 *	118.14 ± 12.93	109.29 ± 12.72 ^#^
TCHO (mg/dL)	124.14 ± 14.3	215.57 ± 21.16 **	215.00 ± 35.72	201.29 ± 37.75
LDLC (mg/dL)	22.00 ± 4.26	59.74 ± 5.24 **	39.53 ± 10.24 ^##^	33.33 ± 20.71 ^##^
HDLC (mg/dL)	83.86 ± 9.19	131.43 ± 18.18 **	157.43 ± 25.83	150.86 ± 25.39
HTR (%)	67.63 ± 2.14	60.77 ± 3.35 **	70.80 ± 9.67	69.61 ± 8.23

Results shown are mean ± SD (*n* = 5). Data are statistically represented with * = significantly different from the NC group, and ^#^ = significantly different from PM group; * and ^#^ *p* < 0.05, ** and ^##^ *p* < 0.01.

**Table 4:** Effect of 80% ethanolic extract from Gimcheon 1ho (GC) cultivar walnut (*Juglans regia*) on organ weights.

Group	NC	HFD	GC20	GC50
Brain (g)	0.36 ± 0.04	0.36 ± 0.03	0.36 ± 0.04	0.35 ± 0.02
Liver (g)	1.28 ± 0.27	2.67 ± 0.31 **	1.70 ± 0.16 ^##^	1.54 ± 0.34 ^##^
Hepatic lipid (mg/g)	6.83 ± 0.13	19.58 ± 2.93 **	15.28 ± 0.86 ^#^	10.28 ± 2.75 ^##^
Perirenal WAT fat (g)	0.12 ± 0.10	0.35 ± 0.11 **	0.40 ± 0.06	0.40 ± 0.07
Retroperitoneal WAT fat (g)	0.29 ± 0.19	1.23 ± 0.33 **	0.71 ± 0.28 ^##^	0.75 ± 0.17 ^##^
Epididymal WAT fat (g)	1.25 ± 0.39	2.22 ± 0.54 **	1.65 ± 0.49 ^#^	0.94 ± 0.73 ^##^
Mesenteric WAT fat (g)	0.25 ± 0.13	0.90 ± 0.22 **	0.91 ± 0.27	0.95 ± 0.09
Total WAT fat (g)	2.00 ± 0.45	4.39 ± 0.29 **	3.50 ± 0.05 ^#^	3.26 ± 0.40 ^##^

Results shown are mean ± SD (*n* = 5). Data are statistically represented with * = significantly different from the NC group, and ^#^ = significantly different from PM group; ^#^ *p* < 0.05, ** and ^##^ *p* < 0.01.

## Data Availability

The data underlying this article are shared upon reasonable request to the corresponding author.
